# Supplementary material for: Intracellular Energy Variability Modulates Cellular Decision-Making Capacity
Source: Sci Rep. 2019 Dec 27;9:20196. doi: 10.1038/s41598-019-56587-5 (PMC6934696; doi:10.1038/s41598-019-56587-5)
Supplement: Supplementary file 1 — Supplementary Information [file 41598_2019_56587_MOESM1_ESM.pdf]

# Intracellular Energy Variability Modulates Cellular Decision-Making Capacity

**Ryan Kerr**[1], **Sara Jabbari**[1], **and Iain G. Johnston**[2,3,*]

[1]School of Mathematics & Institute of Microbiology and Infection, University of Birmingham, United Kingdom
[2]Faculty of Mathematics and Natural Sciences, University of Bergen, Norway
[3]Alan Turing Institute, London, United Kingdom
[*]Correspondence to iain.johnston@uib.no

## ABSTRACT

# Supplementary Information

### Modelling the Energy Parameter Through a Linear Function

The parameter $\lambda$ was initially modelled by the sigmoidal curve in Equation 4. Modelling the ATP-dependent modulatory term through a linear relationship, $\lambda = A^*$ (Fig. S1), whilst fixing $\theta_a$, $\theta_b$, $n$ and $k$ at their default value, shows qualitatively similar behaviour compared to when $\lambda(A^*)$ is sigmoidal (Fig. 4). In contrast we observe that significantly lower $A^*$ thresholds are required for multiple stable steady states, increased parameter sets with 4 stable attractors and there exist more parameter sets with re-entrant behaviour.

The choice of function to model $\lambda$ therefore has an effect on the qualitative behaviour, but the number of stable attractors still increases as $A^*$ increases. Biologically this again displays an increased decision-making landscape for a cell as intracellular energy budget increases, as observed for sigmoidal $\lambda$.

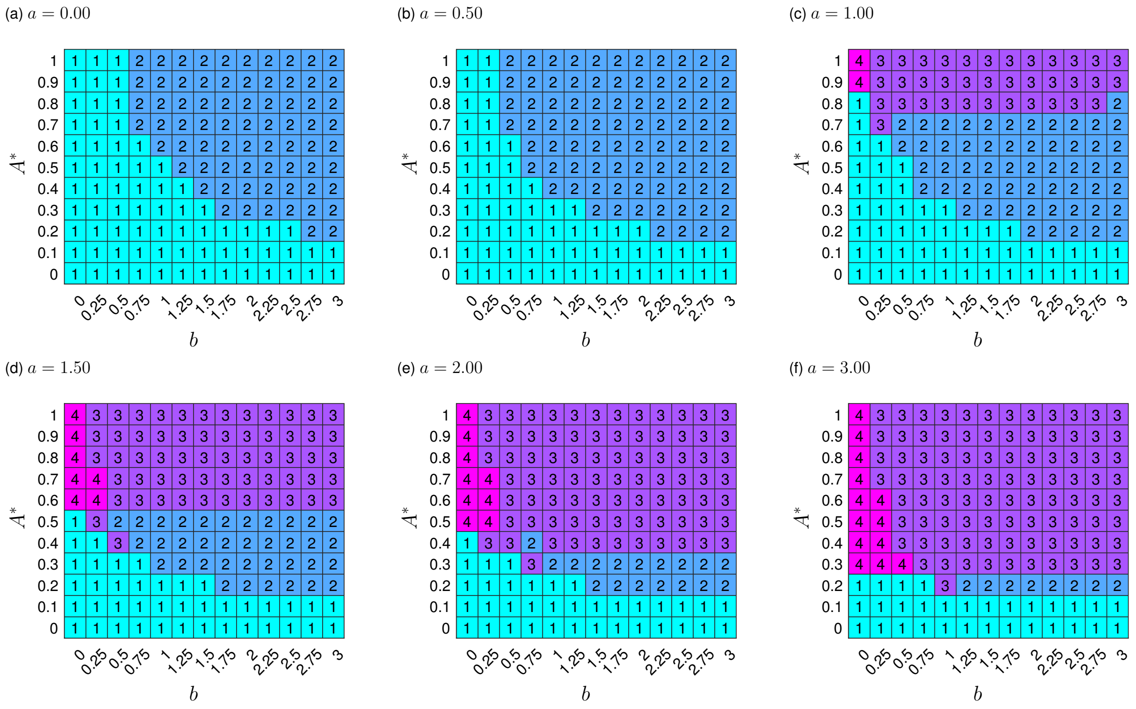


**Supplementary Figure S1. Linear energy function displays similar qualitative decision-making landscapes to the default case.** Panels (a)-(f) display heatmaps for 6 increasing values of $a$, when $\lambda = A^*$. Each panel exhibits the number of stable steady states for combinations of $b \in [0,3]$ and $A^* \in [0,1]$ with all remaining parameters fixed at their default values.

## Sigmoidal Energy Dependent Parameter

The energy dependent parameter $\lambda(A^*)$, modelled through Equation 4 with $s_1 = 16$ and $s_2 = -8$, is displayed in Fig. S2. Included is a biological example, displaying the known *E. coli* intracellular ATP concentration range ($1.54 \pm 1.22$ mM[1]).

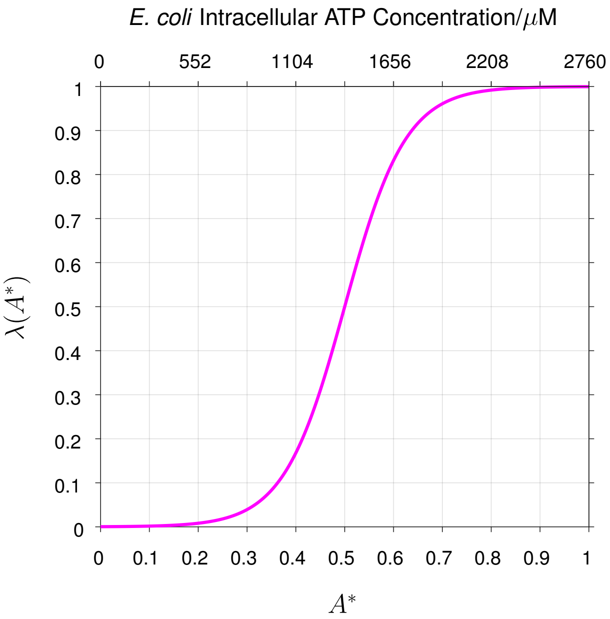


**Supplementary Figure S2. Model relationship between energy availability and gene expression rates.** Sigmoidal curve given by Equation 4, displaying limiting $\lambda$ for small $A^*$ and maximal $\lambda$ as $A^*$ approaches 1. Secondary $x$-axis (top $x$-axis) provides a biological example of the intracellular ATP concentration of *E. coli* with respect to the maximum ATP concentration using known upper and lower bounds[1]. In this example, the lower bound of *E. coli* intracellular ATP concentration is 320 $\mu$M, corresponding to small $\lambda$.

## Bifurcation Diagram Example

Figure S3 displays the bifurcation diagram for default values and varying $A^*$. Inset figures show time-dependent trajectories towards stable attractors for fixed $A^*$ values.

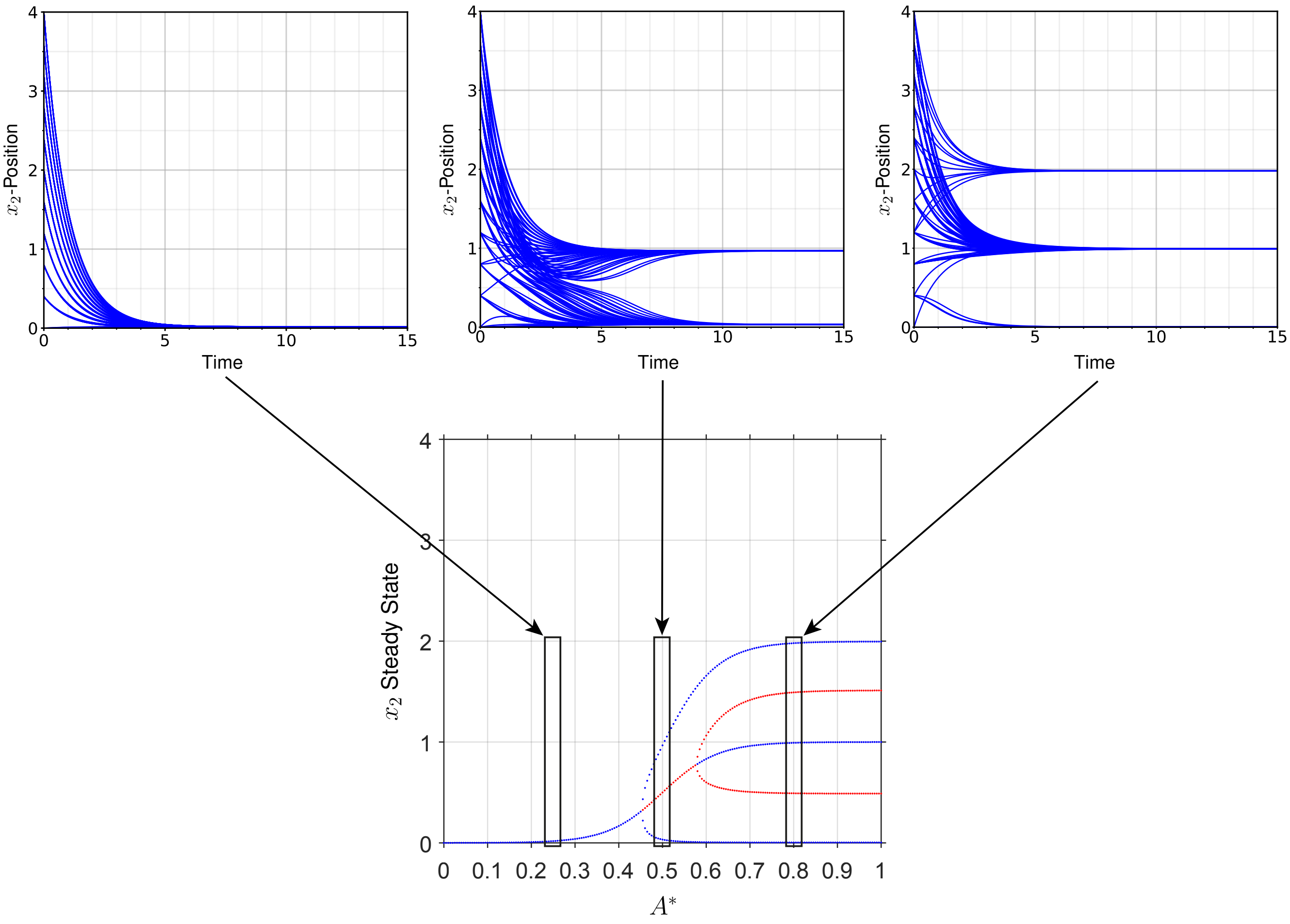


**Supplementary Figure S3. Increasing energy levels support more distinct attractors.** Bifurcation diagram for the default parameter set, displaying stable steady states (blue circles) and unstable steady states (red circles) for $A^* \in [0, 1]$ in steps of size $5 \times 10^{-3}$. Insets show behaviour of time-dependent simulations with multiple initial conditions at specified $A^*$ values. Time-dependent examples are displayed for $A^* = 0.25, 0.5$ and $0.8$ to show examples of the behaviour included in the bifurcation diagram.

## Re-Entrant Behaviour

Figures S4 and S5 show the re-entrant behaviour displayed in Figs. 2(d) and (f), respectively. Re-entrant behaviour contained in Figs. 4(c)-(d) are presented in Fig. S6 (varying $A^*$) and S7 (varying $b$), respectively.

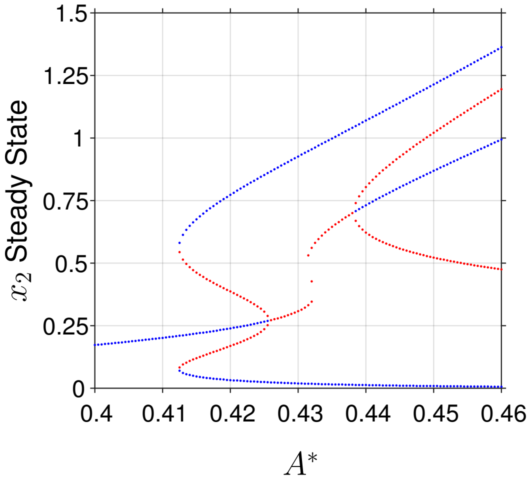


**Supplementary Figure S4. Re-entrant behaviour in decision making options.** Close-up view of Figure 2(d) over $A^* \in [0.4, 0.46]$ in steps of size $5 \times 10^{-4}$. The figure displays stable steady states (blue circles) and unstable steady states (red circles). The number of stable steady states transitions from 1 to 3, then 3 to 2 and finally 2 to 3 in a small region of $A^*$.

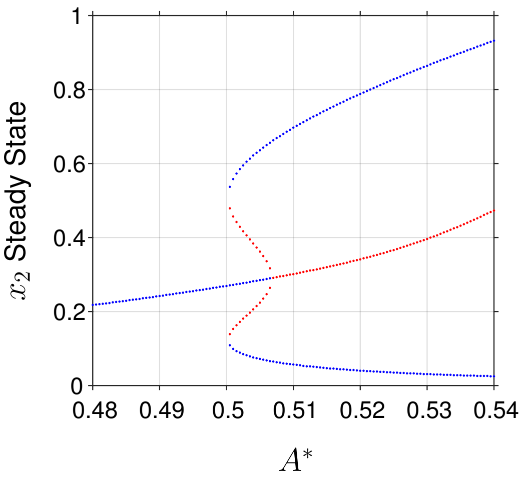


**Supplementary Figure S5. Destabilisation of central attractor in re-entrant behaviour.** Close-up view of Figure 2(f) for $A^* \in [0.45, 0.5]$ in steps of size $5 \times 10^{-4}$. The figure displays stable steady states (blue circles) and unstable steady states (red circles). The number of stable steady states transitions from 1 to 3, then 3 to 2 in the small region of $A^* \in [0.477, 0.484]$.

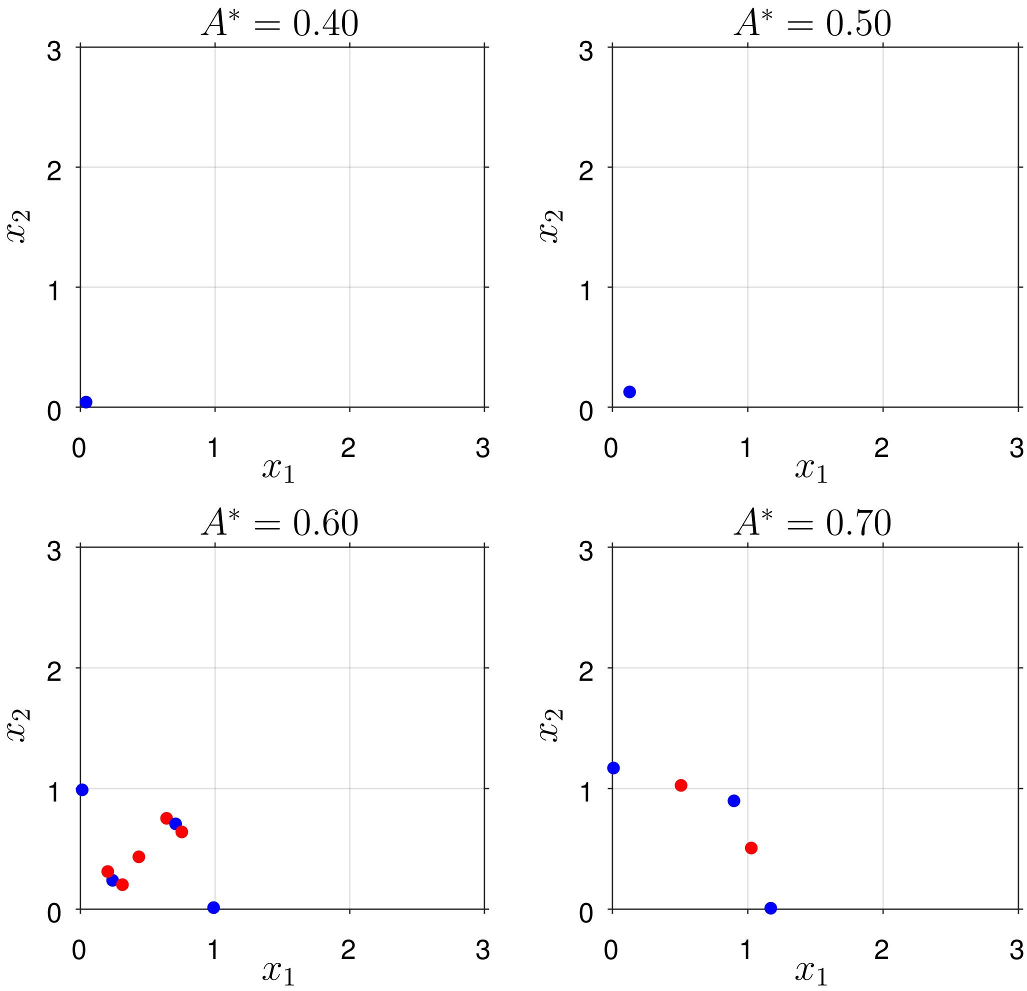


**Supplementary Figure S6. Re-entrant behaviour showing destabilisation of the 'near-zero' attractor.** Array displays stable (blue) and unstable (red) states for $a = 1$, $b = 0.25$ and $A^* \in [0.4, 0.7]$ in Figure 4(c). Increasing energy develops 4 stable attractors which decreases to 3 as $A^*$ increases. All remaining parameters are at their default value.

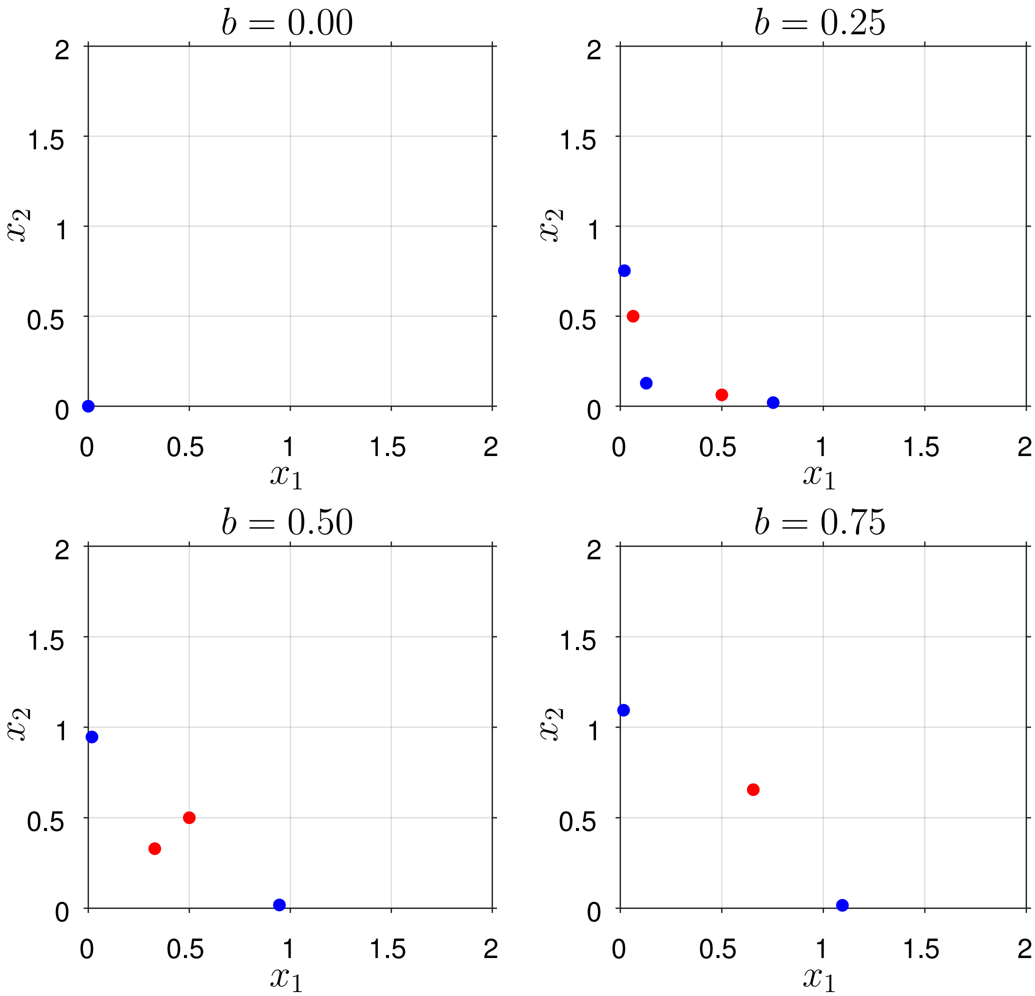


**Supplementary Figure S7. Re-entrant behaviour showing destabilisation of the central attractor.** Array displays stable (blue) and unstable (red) states for $a = 1.5$, $A^* = 0.5$ and $b \in [0, 0.75]$ in Figure 4(d). Increasing energy develops 3 stable attractors from the initial single stable state which decreases to 2 as $b$ increases. All remaining parameters are at their default value.

### Effects on the Decision-Making Landscape When Reducing $n$

Decreasing the Hill function coefficient, $n$, from the default value ($n = 4$) to $n = 1$ is displayed in Figs. S8-S10 and exhibit the changes in qualitative behaviour as the amount of cooperativity is reduced.

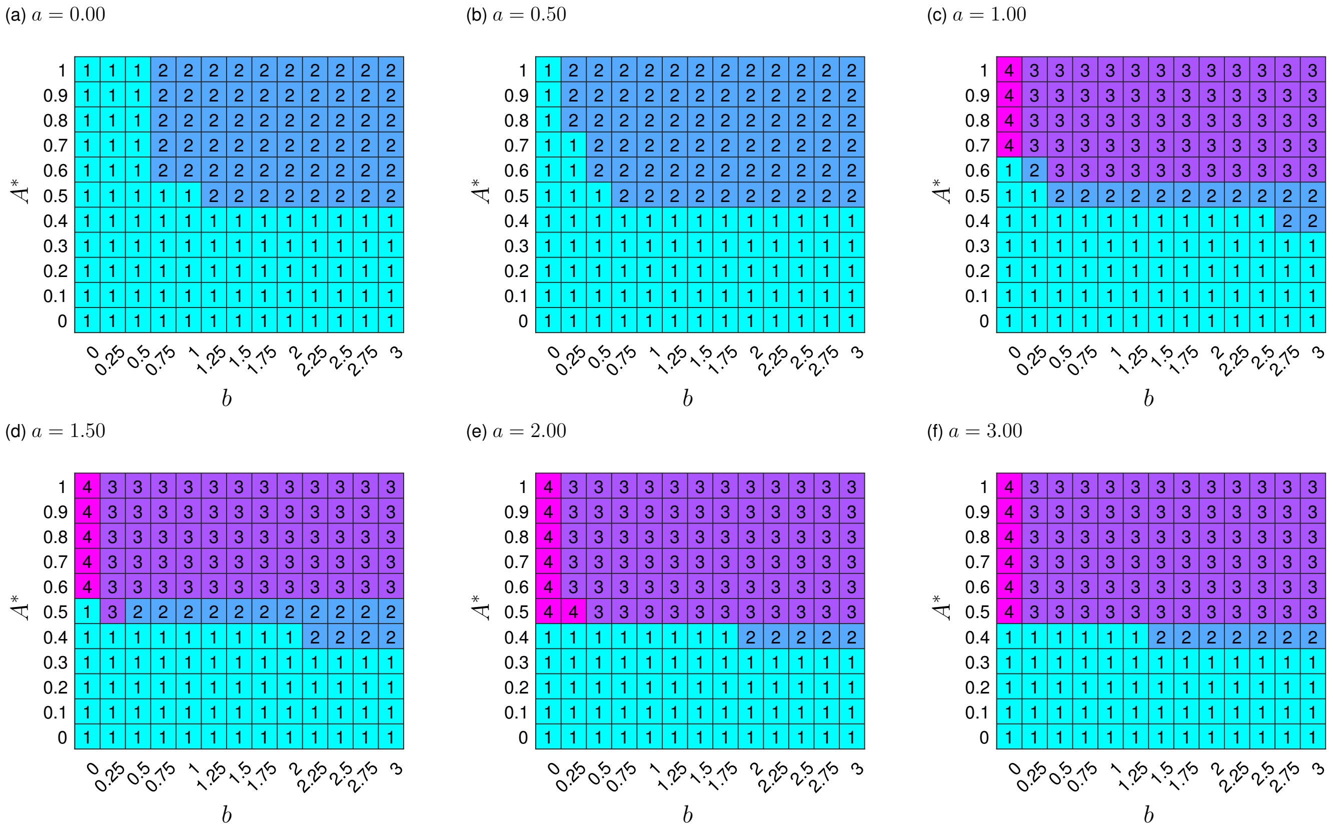

**Supplementary Figure S8. Reduced Hill coefficient value ($n = 3$) displays similar qualitative decision-making landscapes to the default value ($n = 4$).** Panels (a)-(f) display heatmaps for 6 increasing values of $a$, when $n = 3$. Each panel exhibits the number of stable steady states for combinations of $b \in [0,3]$ and $A^* \in [0,1]$, with all remaining parameters fixed at their default values.

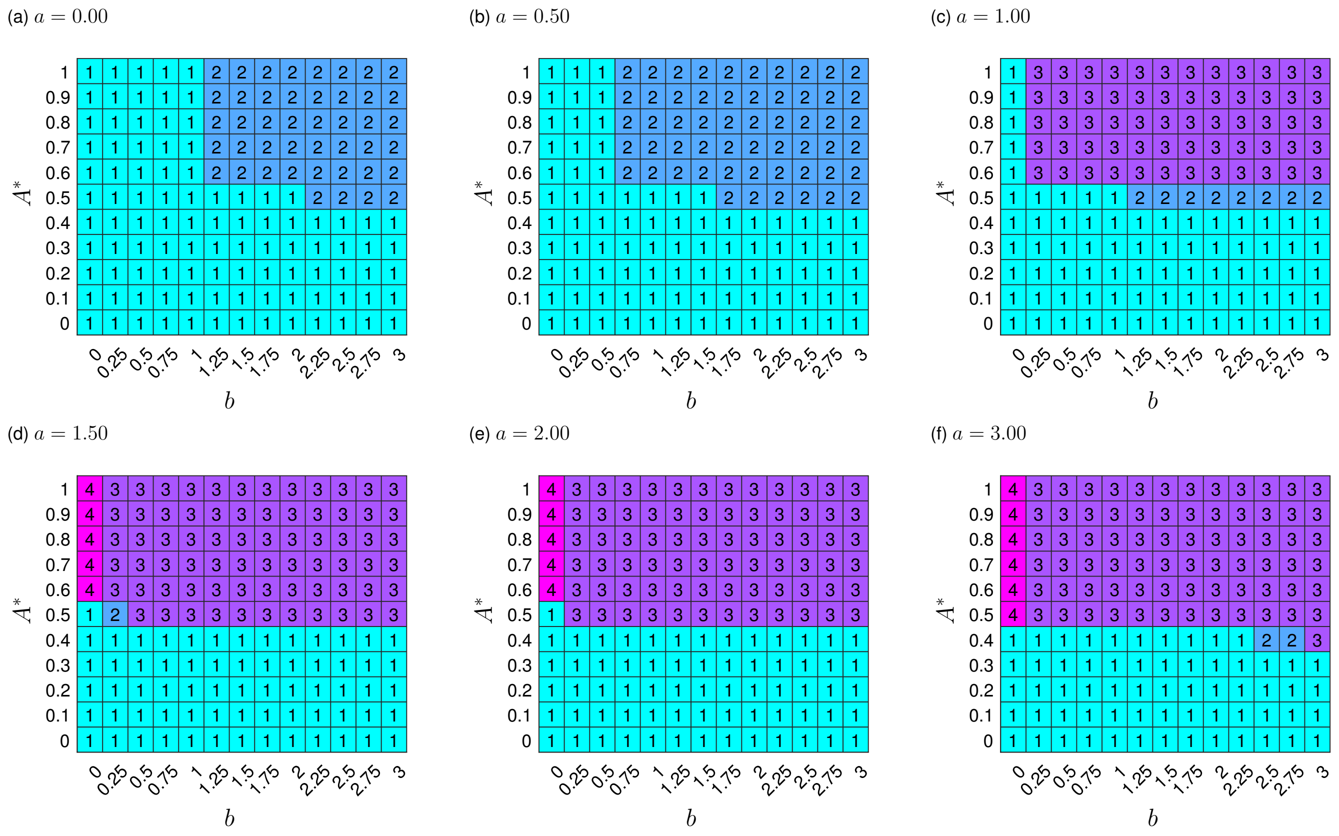

**Supplementary Figure S9. Hill coefficient $n = 2$ displays simplified qualitative decision-making landscapes to the default value ($n = 4$).** Panels (a)-(f) display heatmaps for 6 increasing values of $a$, when $n = 2$. Each panel exhibits the number of stable steady states for combinations of $b \in [0,3]$ and $A^* \in [0,1]$, with all remaining parameters fixed at their default values.

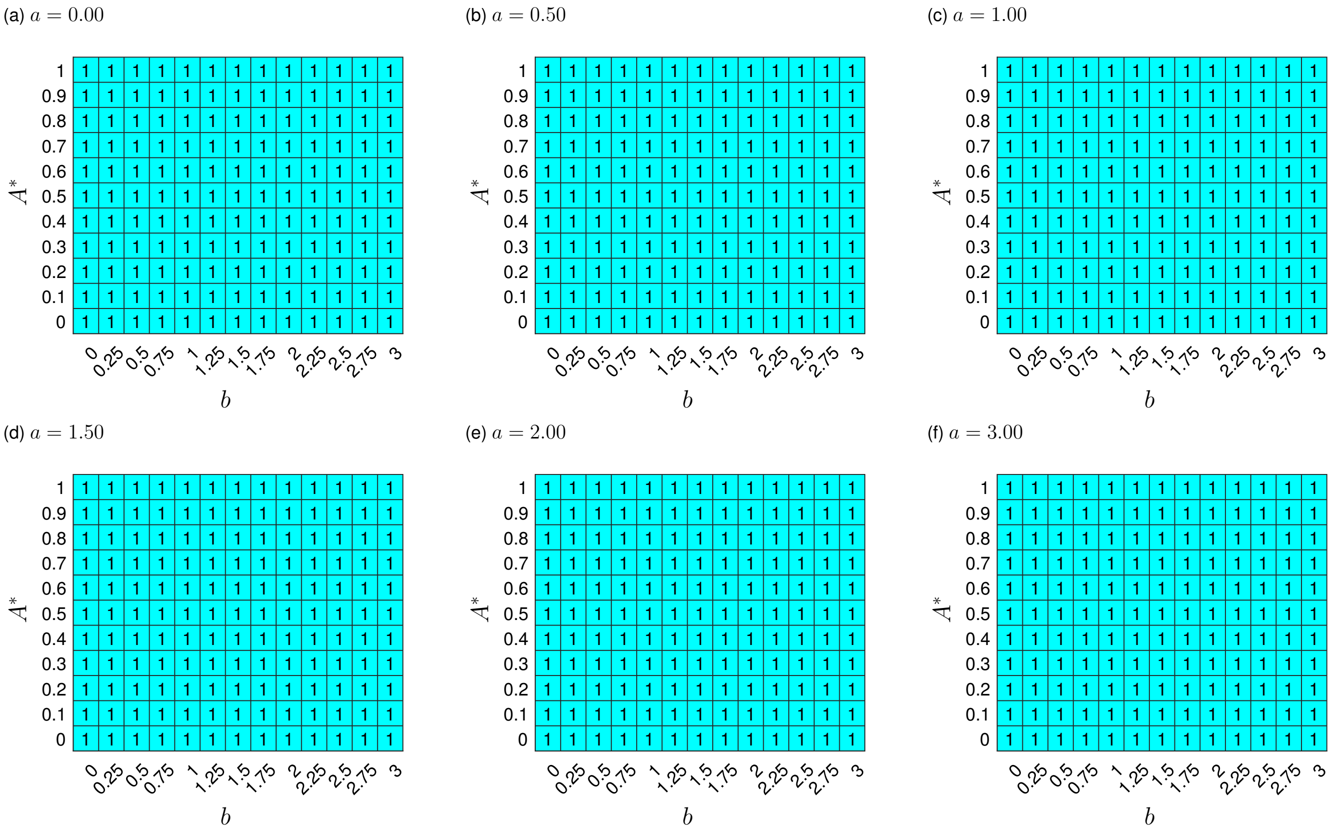


**Supplementary Figure S10. Non-cooperative binding limits the decision-making landscape.** Panels (a)-(f) display heatmaps for 6 increasing values of $a$, when $n = 1$. Each panel displays the number of stable steady states when spanning $b \in [0,3]$ and $A^* \in [0,1]$, with all remaining parameters fixed at their default values.

## Regulatory Protein Binding Strength Heatmaps

Figures S11(a)-(b) show the effect of varying activator and repressor protein binding strength, respectively.

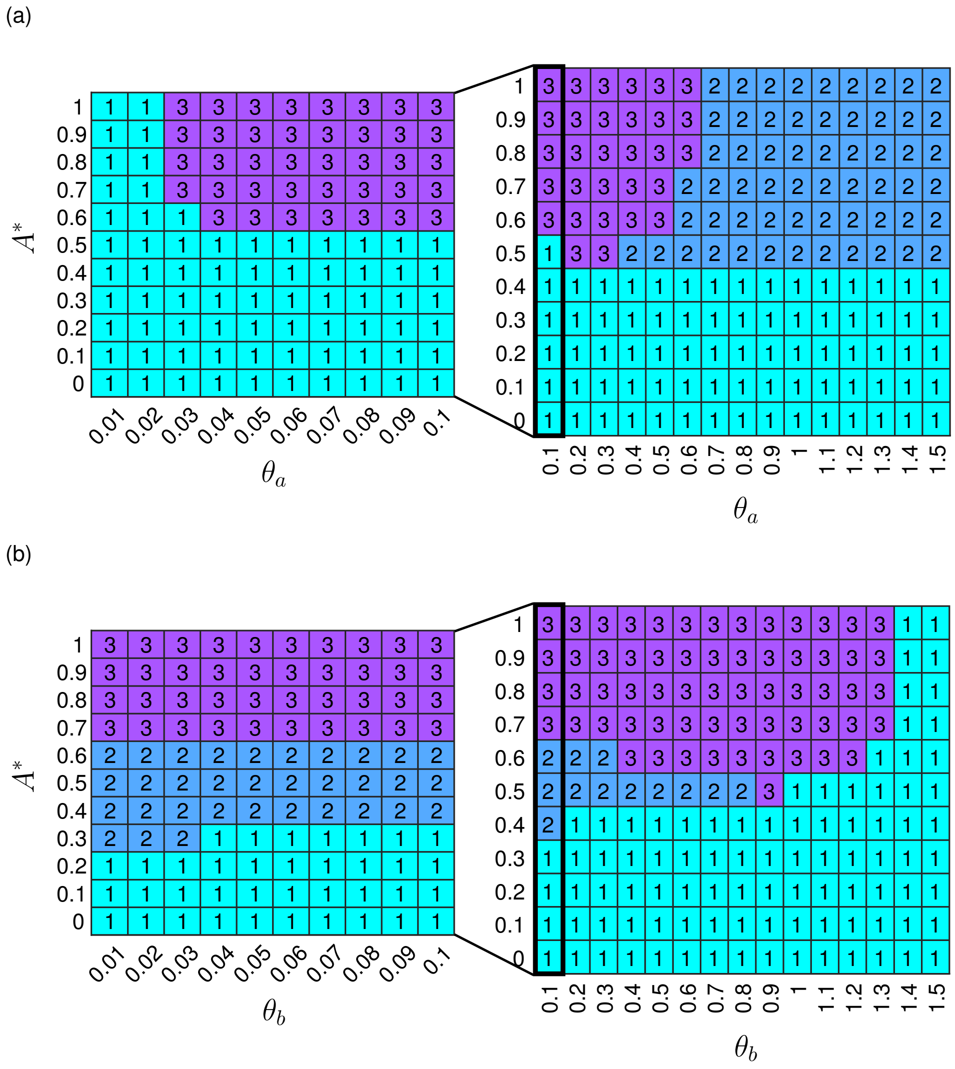


**Supplementary Figure S11. Strength of binding limits the decision-making landscape.** Main panels display the number of stable states for (a) $\theta_a$, (b) $\theta_b \in [0.1, 1.5]$ and $A^* \in [0, 1]$. Inset sub-panels display stable attractor behaviour for $\theta_a$, $\theta_b \in [0.01, 0.1]$. Each panel contains colour coordinated number of stable steady states: 1 (turquoise); 2 (blue); 3 (purple). Remaining parameters are fixed at their default values. Behaviour displayed for $\theta_a = \theta_b = 1.5$ continues for larger values of each parameter.

## External ATP Effects

Figures S12 and S13 show the modified form of $\lambda$ when considering external ATP effects and the impact on the attractor landscape, respectively.

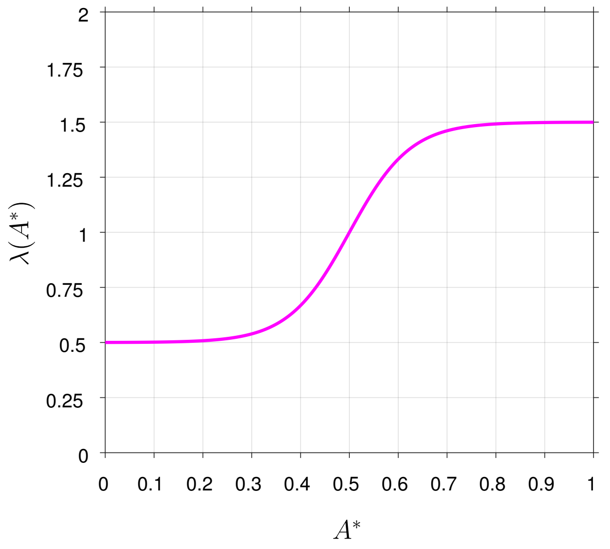


**Supplementary Figure S12. Model relationship between energy availability and gene expression rates with external ATP influences.** Modified $\lambda$ produced by vertically displacing the original sigmoid in Fig. S2

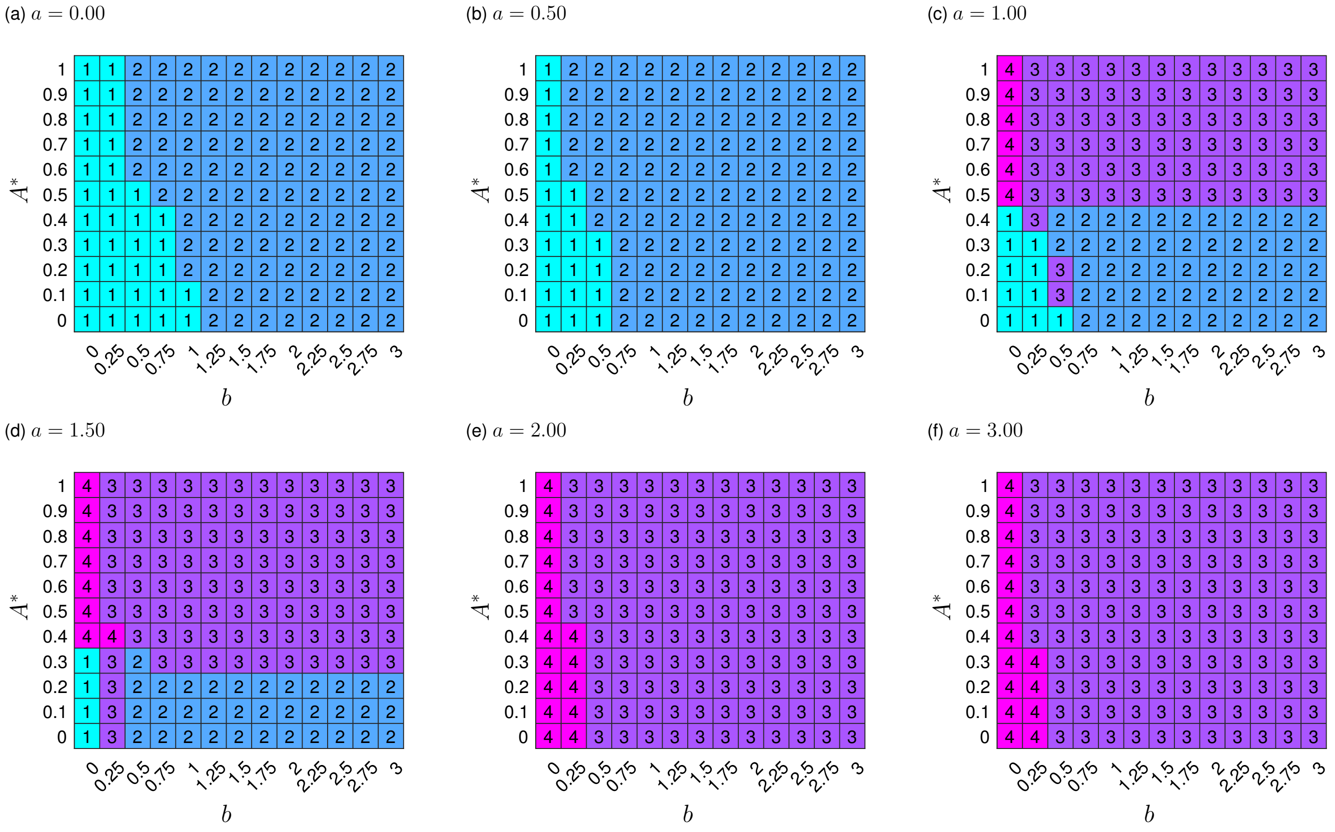

**Supplementary Figure S13. Decision-making landscape variability is observed when considering external ATP influences.** Panels (a)-(f) display heatmaps for 6 increasing values of $a$, when $n = 4$, for the sigmoid in Fig, S12. Each panel exhibits the number of stable steady states for combinations of $b \in [0,3]$ and $A^* \in [0,1]$, with all remaining parameters fixed at their default values.

### Attractor Transition ‘Difficulty’

The minimum distance to transition between extreme attractor states (for landscapes with 2 or more attractors) is displayed in Fig. S14 for $n = 4$, showing how attractor transition ‘difficulty’ develops as energy availability is varied. The position of attractors within the landscape changes with the parameters, explaining the large distances observed and the re-entrant behaviour for transition ‘difficulty’ (see Fig. S14 (c) and (d)) is an effect of the trade-off between increasing attractor distances, the number of attractors and the ease of traversal of the central basin.

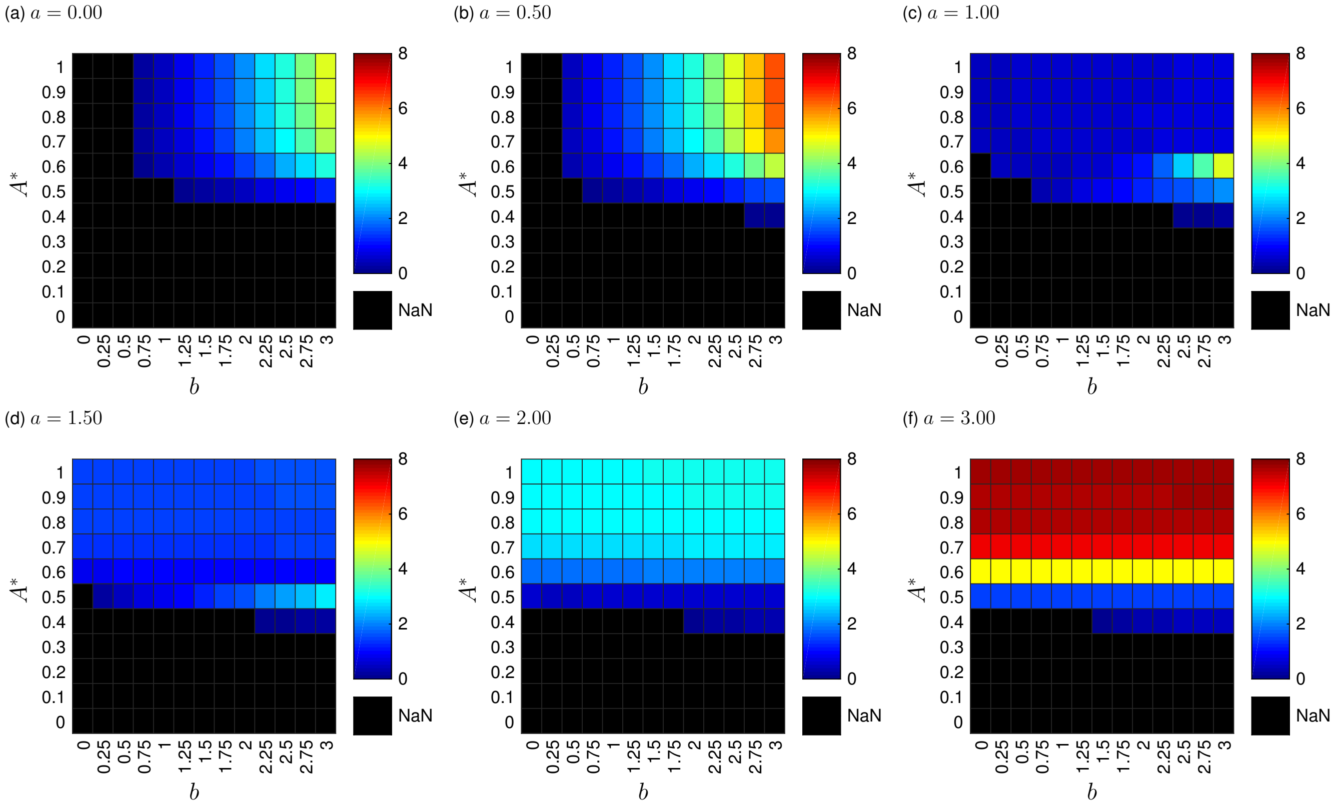


**Supplementary Figure S14. The ability to transition between extreme states becomes harder as energy availability increases.** Panels (a)-(f) display heatmaps for 6 increasing values of $a$, when $n = 4$. Each panel exhibits the minimum distance between stable steady states, representing the ‘difficulty’ of extreme attractor transitions, for combinations of $b \in [0,3]$ and $A^* \in [0,1]$, with all remaining parameters fixed at their default values. The work required to transition ranges from low (dark blue) to high (dark red). For monostable landscapes the metric is not defined and is represented by black in each panel.

## References

1. Yaginuma, H. *et al.* Diversity in atp concentrations in a single bacterial cell population revealed by quantitative single-cell imaging. *Sci. reports* **4**, 6522 (2014).
